# New Insights Into Breast and Chest Wall Asymmetry in the Aesthetic Patient

**DOI:** 10.1093/asjof/ojae080

**Published:** 2024-09-14

**Authors:** Farhad Hafezi, Abbas Kazemi Ashtiani, Mahdokht Azarbakhsh, Jaafari Ali, Soheila Naderi Gharegheshlagh

## Abstract

**Background:**

Asymmetry, a prevalent phenomenon throughout the human body, prompts this retrospective study, in which the authors aim at discerning potential patterns in its manifestation. Building upon our previous investigations in which left-sided chest wall and pelvic bone width surpassed their right-sided counterparts, a hypothesis is formulated suggesting the likelihood of the right breast being narrower and longer compared with the left.

**Objectives:**

Our objective in this study is to investigate the correlation between the left-sidedness phenomenon in the chest wall and breast shape and appearance, with the aim of understanding its potential impact on outcomes in breast aesthetic surgery.

**Methods:**

A random selection of pictures from 600 female patients undergoing various aesthetic breast procedures formed the basis of this study. Exclusion criteria involved the elimination of 254 pictures with nonstandard photography. Horizontal and vertical parameters of breasts and chest walls were measured, and the results on both sides were systematically compared to validate our hypothesis.

**Results:**

On the left side, the chest wall, the distance between the nipple and the midline, and that of the nipple and the anterior axillary line were significantly wider. Vertical measurements, including the sternal notch to the nipple, the clavicle to the nipple, and the clavicle to inferior breast distance, although longer, did not exhibit statistical significance.

**Conclusions:**

Approximately 77% of patients displayed wider breasts and chest walls on the left side. However, this observed asymmetry did not yield a statistically significant impact on the length of the breasts.

**Level of Evidence: 3 (Diagnostic):**

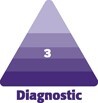

The authors of previous research have explored the intricacies of facial, nasal, and bodily asymmetry. In the initial exploration, they focused on the correlation between facial and nasal skeletal asymmetry.^1^ The authors of subsequent research involving measurements of 42 natural human skulls revealed a wider facial skeleton on the left side.^2^ The authors of further studies extended their research to patients who had undergone rhinoplasty, integrating computed tomography scan measurements of facial skeleton, soft tissue, chest wall, and pelvic bone width.^3^ The authors of this comprehensive analysis identified a significant left-sided dominance in both facial and body measurements, termed as the “left-sidedness phenomenon.”^3^

Evidently, asymmetry in the human body, and possibly in other bilaterian animals, extends beyond internal organs such as the heart, lungs, and abdominal viscera. This nonuniformity appears to be orchestrated by a genetic sequence governing the soft tissue and skeleton throughout the entire body. As a prevailing tendency, this asymmetry is both constant and frequent. Given the observed “left-sidedness phenomenon” on the chest wall, we postulated that the broader left chest wall might exert an influence on breast shape and appearance, potentially impacting outcomes in breast aesthetic surgery. To explore this hypothesis, we conducted an analysis, measuring various dimensions of breasts and chest walls in patient photographs to ascertain whether any significant differences exist between the 2 sides.

## METHODS

A retrospective study was conducted on 600 female patients who underwent aesthetic breast surgery at the clinic of the corresponding author (F.H.) between September 2014 and June 2021. Procedures included breast augmentation, breast reduction, and mastopexy. Inclusion criteria involved females aged 18 to 68 years, with normal breast, chest wall, and spine morphology, regardless of BMI, race, breast characteristics, or breastfeeding history. Exclusion criteria encompassed skeletal deformities, traumatic scars, and previous breast or chest wall operations. We did not exclude other normal breast variations, such as tuberous breasts.

The authors declare that the procedures were followed according to the Declaration of Helsinki.

## RESULTS

**Figure 1.**
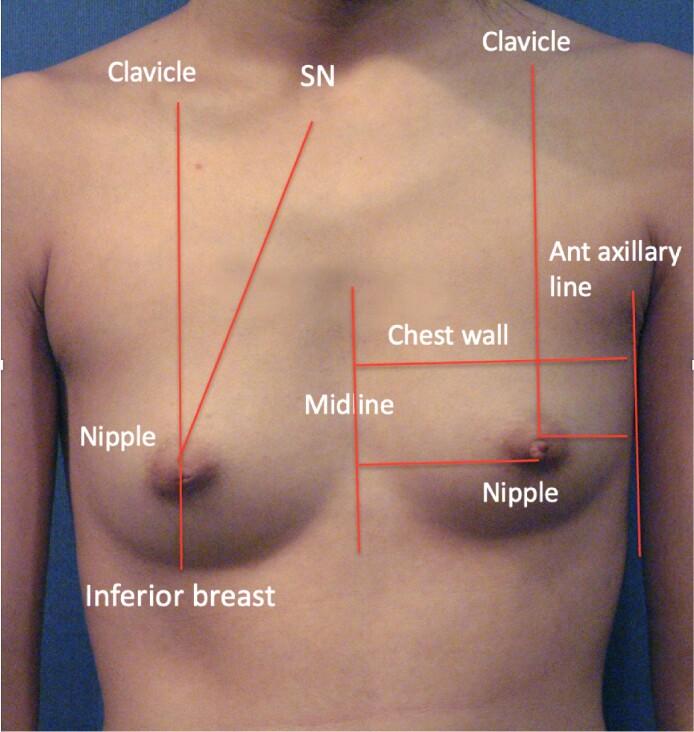
Measured dimensions of the breasts and chest wall.

Photographs of all 600 female patients were analyzed, with exclusions (254) based on improper posture and inaccuracies in fixed anatomical points. The remaining 346 patients underwent measurements of defined fixed points on the right and left breasts and chest wall using Image J software by a blindfolded physician (Figure 1). The age range of patients was 18 to 62 years, with an average age of 34 years. The data were analyzed using SPSS software (IBM, Armonk, NY), and Kappa coefficients were employed for agreement comparisons. Negative Kappa values indicated an agreement worse than chance, suggesting no consistency between observation and theory. Our findings suggest that the breasts and chest wall exhibit greater width on the left side (Table 1). However, regarding breast length, no findings were reported. In essence, our hypothesis is partially substantiated in the context of width but remains inconclusive for breast length (Table 2). Further research with refined methodologies is warranted to provide more definitive insights into these asymmetries.

**Table 1.** Left- and Right-Side Width Frequency Differences

Variable	Kappa statistic	Asymptotic SE	Approximate *P*-value
Chest wall wideness	0.166	0.016	<.001
Nipple to midline distance	0.080	0.017	<.001
Anterior axillary to nipple	0.080	0.017	<.001

Significant differences in width frequency were observed between the left and the right sides (*P* < .001), indicating more frequent width on the left side.

**Table 2.** Vertical Distances Comparison

Variable	Kappa statistic	Asymptotic SE	Approximate *P*-value
SN-N	−0.045	0.018	.011
Clavicle nipple distance	−0.022	0.018	.212
Clavicle-inf. breast distance	−0.036	0.018	.039

Vertical distances between the sternal notch to the nipple, mid-clavicle points to the nipple, and mid-clavicle point to the inferior mammary line were not significantly longer on the right side (*P* < .011).

## DISCUSSION

Reviewing classic embryologic references, bilateral asymmetry initiates as part of the gastrula stage of embryonic formation and continues as the fetus grows in the uterus. In humans, the cryptic gene has been demonstrated to be responsible for the establishment of the L–R axis.^4^ This physiologic phenomenon occurs early in development in a structure called the “left–right organizer,” leading to the activation of different signaling transductions on the left and right sides of the embryo.

The “morphogen” hypothesis has been proposed to explain the morphogenesis behavior of cells and the function of nodal flow in human embryos, characterized by a clockwise rotatory movement of motile cilia that transports a morphogen toward the left side of the ventral node.^5^ It is considered a fundamental pattern of human development (Video). Although left–right asymmetry is traditionally associated with internal organs, such as the tilt of the heart, the number of lung lobes, and the position of the stomach and spleen, it is more logical to consider that this unique physiological role extends to various parts of the body. The human embryology and developmental biology literature generally focuses on asymmetry in internal organs, often overlooking other body formations. However, our assumption is to expand this phenomenon to the surface area and musculoskeletal system, including the chest wall and breasts.

There are various congenital and developmental anomalies of the chest wall and spine that can lead to asymmetry in breast width, height, and projection. Structural deformities such as pectus excavatum and pectus carinatum and spine deformities such as scoliosis and kyphosis may result in noticeable breast asymmetries. In multiple studies, it has been observed that ∼10.6% to 36% of breast augmentation candidates suffer from various chest wall deformities.^6,7^ Developmental anomalies such as Poland syndrome require special consideration to achieve approximate symmetry.

Although the chest wall contour may change with age, Hirsch and Brody found that these changes occur simultaneously, and asymmetry is not related to age.^8^ In this study, we are concerned with minimal chest wall and breast asymmetries that may be overlooked by both patients and surgeons. Furthermore, these subtle yet significant right–left imbalances may interfere with preoperative calculations and surgical plans. Appreciating and recognizing these disparities can assist surgeons in planning the operative technique for aesthetic breast surgery and selecting the appropriate prosthesis size. Additionally, preoperative patient education about the details of their asymmetries is essential. This education helps patients understand their inherent skeletal and chest wall unevenness and the limitations of the surgeon's ability to achieve impeccable symmetry. Geometrical symmetry is unattainable, and therefore, patients should be informed about this fact, providing them with more realistic expectations and satisfaction regarding the outcome of their operation.

Although there is an apparent lack of information on breast and chest wall asymmetries,^9^ researchers in their reports indicate a significant prevalence of breast asymmetry among the normal population seeking breast augmentation. Liu et al reported asymmetry in 73% of nipple positions, 95% of breast shapes, and 38% of anterior chest wall projections.^10,11^ In another study, researchers measured female’s breasts in the normal population and found an 81.7% difference between right and left breasts, 59.6% from nipple to inframammary fold, and 81.2% from sternal notch to nipple. They concluded that some degree of asymmetry was present in all of their patients.^7,8,10-12^ Losken et al studied different dimensions of the breast in 87 normal females and found that, on average, the left breast was bigger compared with the right side.^12^ Some authors have developed software, the Breast Analyzing Tool, which may help detect asymmetries more accurately.^13^ Chest wall asymmetry occurs not only in width and height but also in the projection of the chest wall cavity, with curvature differences in the ribs between the right and the left sides, 36%.^7,8^

This oversight is probably attributed to the minimal and imperceptible disparity of the skin and musculoskeletal systems. This may be the cause of patient dissatisfaction after breast augmentation, because it can result in asymmetric upper pole fullness. Postoperatively, the size and position of the breasts on the chest wall are of crucial concern.^14^

Geographical distribution and ethnicity have no effect on the occurrence of this anatomic variance. This asymmetry is more pronounced in females with hypertrophic and pendulous breasts.^9,15^ It appears that measuring breast length is easier in pendulous breasts than in smaller-sized breasts. However, in patients who are candidates for breast augmentation and have insufficient breast tissue adhering to the chest wall, this asymmetry is less appreciable, particularly when measuring vertical dimensions. In thin patients with a lack of fatty tissue around the breast and axilla, measuring the chest wall width is more accurate. These observations could be the cause of minor errors in accurately measuring breast and chest wall dimensions, potentially biasing this study.

Knowledge and understanding of the rules of chest wall and breast asymmetry can help in selecting more precise breast implants that conform to the upper trunk anatomy, aligning with the patient's breast footprint. According to the above findings, the right chest wall and breast width are narrower than the left, suggesting that it is logical to select a wider prosthesis with a lower projection on the right and a prosthesis with a higher projection on the left, or at the very least, inform the patient about this unevenness. However, using a wide and large implant in a narrow chest wall may result in an unnatural appearance and visibility of the prosthesis. Therefore, the placement of different-sized prostheses should be approached cautiously. Mallucci and Branford also recommended, in their paper, the use of asymmetric prostheses with different sizes and shapes to correct asymmetric chest wall and breasts.^16^

**Figure 2.**
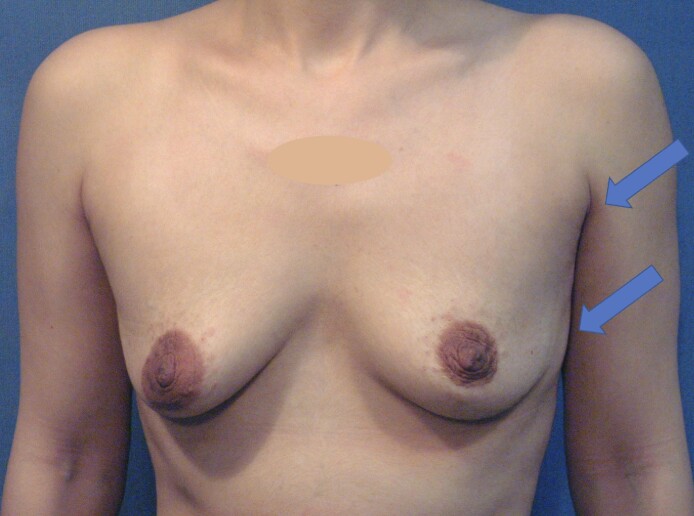
A 24-year-old female has been referred for breast augmentation. In the front view, there is noticeable asymmetry, with the left side chest wall and breast appearing larger in width.

**Figure 3.**
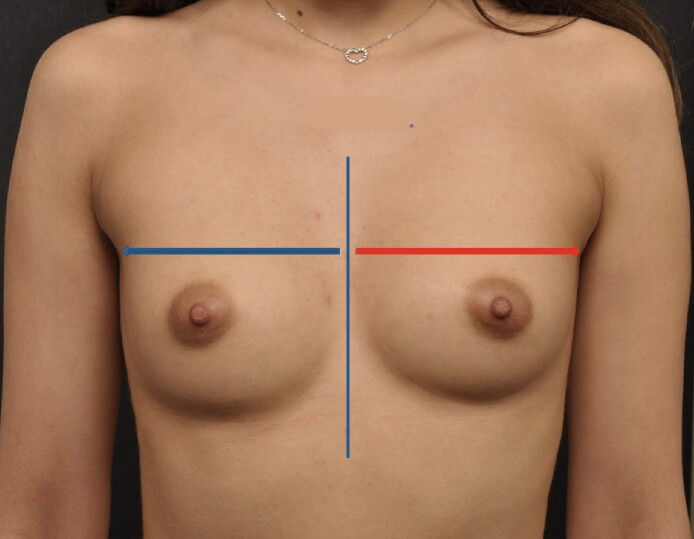
A 32-year-old female candidate is being considered for breast augmentation. It is noted that even though she has small breasts, there is more soft tissue present on the left side, along with a wider chest wall skeleton.

Observing patient pictures indicates that axillary soft tissue and fat content are more prominent on the left side, even in thinner patients in need of breast augmentation or male patients (Figure 2). Removing more axillary fat on the left side, either surgically or by liposuction, may result in greater symmetry. Most patients seeking breast reduction surgeries may benefit from these procedures (Figure 3).

The insights from the above findings are not only valuable in the planning of aesthetic breast surgery but also have potential industrial applications. In the clothing industry, knowledge of body asymmetry rules can assist clothes designers in creating better-fitting garments by tailoring shoulders unequally (Video).

It is important to acknowledge that the inaccuracies in standard fixed points and inevitable measuring errors have introduced bias and limitations to this study. Moving forward, a prospective study, as opposed to a retrospective one, coupled with more precise, real-time breast measurements, and the integration of 3-dimensional photography could significantly enhance the accuracy and reliability of our findings. This reflection underscores the importance of ongoing research and methodological refinement to advance our understanding and improve outcomes in breast augmentation procedures.

The observed rate of 30% dissatisfaction among patients and the need for revisional operations in a subset of breast augmentation cases can be attributed primarily to the oversight of preexisting breast or chest wall asymmetry (83% prevalence).^9^ Enhancing patient education and obtaining informed consent regarding potential variations between the right and the left breasts, as well as chest wall deformities, emerges as a crucial strategy to heighten patient satisfaction and diminish the need for revisions.^17^

## CONCLUSIONS

The authors of the present study have provided new insights into the relationship between breast and chest wall asymmetry, as well as its potential implications for breast aesthetic surgery. Our findings indicate that the breasts and chest wall exhibit a consistent left-sided dominance in terms of width, with a statistically significant difference observed between the 2 sides. This supports our hypothesis that the broader left chest wall may influence breast shape and appearance. However, our investigation failed to uncover a significant difference in breast length between the 2 sides, leaving the relationship between breast length and left-sidedness inconclusive. This lack of distinction could be attributed to challenges in identifying precise fixed points in vertical dimensions and may necessitate further prospective measurements for clarification.
